# Factors affecting survivability of local Rohilkhand goats under organized farm

**DOI:** 10.14202/vetworld.2015.1215-1218

**Published:** 2015-10-22

**Authors:** D. Upadhyay, B. H. M. Patel, S. Sahu, G. K. Gaur, M. Singh

**Affiliations:** 1 Livestock Production and Management Section, Indian Veterinary Research Institute, Izatnagar, Bareilly - 243 122, Uttar Pradesh, India; 2 Department of Livestock Production Management, Lala Lajpat Rai University of Veterinary & Animal Sciences, Hisar, Haryana, India

**Keywords:** digestive disease, goat, mortality, Rohilkhand

## Abstract

**Aim:**

To study the pattern of mortality as affected by age, season and various diseases in local goats of Rohilkhand region maintained at the Indian Veterinary Research Institute, Bareilly.

**Materials and Methods:**

Post-mortem records of 12 years (2000-01 to 2011-12) were used, and total 243 mortality data were collected and analyzed. The causes of mortality were classified into seven major classes viz. digestive disorders, respiratory disorders, cardiovascular disorders, musculoskeletal disorder, parasitic disorders, mixed disorders (combination of digestive, respiratory, parasitic, and cardiovascular disorders) and miscellaneous disorders (cold, hypoglycemia, emaciation, endometritis, traumatic injury, etc.).

**Results:**

The average mortality was 10.93%. The overall mortality was more during rainy season followed by winter and summer season. The mortality in 4-6 months of age was high (2.52%) followed by 0-1 month (2.34%) and 2-3 months (1.35%). The average mortality among adult age groups (>12 months) was 3.42%. The mortality showed declining trend with the advancement of age up to 3 months and then again increased in 4-6 months age group. The digestive diseases (3.51%) followed by respiratory diseases (1.89%) and parasitic diseases (1.48%) contributed major share to the total mortality occurred and the remaining disorders were of lesser significance in causing death in goats. There is significant (p<0.01; χ^2^=55.62) association between year with season and age with the season (p<0.05, χ^2^=16.083) found in the present study.

**Conclusion:**

This study confirms that overall mortality rate averaged 10.93% (ranged between 1.10% and 25.56%) over 12 years under semi-intensive farm condition. It was generally higher in rainy season. The mortality remains higher in kids particularly under 1 month of age. The digestive diseases contributed major share to overall mortality.

## Introduction

More than 95% of the goat population is found in developing countries [1]. India possesses 135.17 million goats [2] and around 26.4% of total livestock in the country, ranking second in goat population of the world. Goat contributes 3.7% (4.8 MT) of the India’s total milk (127.90 MT) production [3]. Goat meat is chosen irrespective of religion or culture; therefore, goat has been designated as the national meat animal in India [4]. The productivity of goats under traditional production system is very low [5].

Commercial goat production is gaining momentum, because of good economic prospects of goat rearing under the intensive and semi-intensive system. Under organized farm mortality due to one or other reason is the matter of concern for producers because it is directly linked with economic loss. Kumar [6], reported estimated losses due to diseases in goats were 23.22% of net returns and 5.21% of gross returns. Economic losses due to mortality caused by various diseases in goats have been a major constraint in the traditional flocks [7]. The disease risk further increases when goats are maintained in large flocks under the intensive system. Several studies [7,8] have shown that on an average 20% of kids and 10% of adult goats die each year. Therefore, the mortality pattern of the farm should be known in order to decide the different managemental practices. To make the goat farming profitable, in addition to strong managemental practices, the herd should be made free from diseases and other health problems.

In the present investigation mortality pattern of local goats of Rohilkhand region was studied. These local goats have been purchased and maintained to characterize and document at the institute since 1997. It is a small sized black goat which looks similar to Black Bengal. The Rohilkhand region has a population of 11.12 lakhs goats out of total population of 14.79 million [3]. Therefore, an attempt has been made to document the mortality pattern over last decade in the experimental goat herd.

## Materials and Methods

### Ethical approval

The experiments on animals were approved by Institutional Animal Ethics Committee of Indian Veterinary Research Instittue (IVRI).

### Animals

Total of 243 mortality records of Rohilkhand local goats, belonging to the experimental herd maintained at institutional sheep and goat farm, IVRI, Izatnagar, Bareilly, for 12 years (2000-01 to 2011-12) were analyzed. The flock used for the study was purchased from animal markets *Rithora* and *Devchara* in the Bareilly district during the year 1997. The goats brought to these markets are the representative of the Rohilkhand region as they are known animal markets of the district. In the beginning 50 nondescript local goats were purchased which are having black coat color with good physical conformity. This base population was continuously bred. The offspring with off color and poor production performance were culled annually. Further, 31 goats have been inducted to the main population during March 2007. Breeding males have been inducted at different interval of time in order to prevent the inbreeding. At given point of time, the average of 150-200 goats was maintained at farm.

### Managemental practices

All the animals were housed in separate sheds and each attached with open paddock, which would allow the animals to loiter freely. Cultivated green fodders (maize/berseem/oat) and water were always available to the experimental animals in sufficient quantity during the course of study. The first fodder of the day was supplied at around 10.30 AM and thereafter, fodder was supplied as and when the feed bunk was found empty. Kids were kept with their mother’s day and night except during some part of the day. Kids were provided with good kid starter (concentrate) along with succulent green fodder when the kids were 3-4 weeks of age. Weaning was practiced at the age of 3 months. Routinely, dead goats were sent to post-mortem (PM) in the pathology division.

### Experimental design

Data on number of animals died were classified as per their respective year of death, season of death, age and sex of animal and cause of death as per PM finding. The season-wise mortality was divided into three seasons, i.e., summer season (March to June), rainy season (July to October) and winter season (November to February). Age-wise mortality was classified into different age groups *viz*., 0-1 month, 2-3 months, 4-6 months, 7-12 months, and adult (>12 months age). The causes of mortality based on PM findings were classified into seven major classes *viz*. digestive disorders, respiratory disorders, cardiovascular disorders, musculoskeletal disorder, parasitic disorders, mixed disorders (combination of digestive, respiratory, parasitic, and cardiovascular disorders) and miscellaneous disorders (cold, hypoglycemia, emaciation, endo-metritis, traumatic injury, etc.).

### Statistical analysis

For calculating percent mortality rate and its distribution pattern, the number of animals died in each year to average herd strength in different years were considered. The association of year and season of death, age, sex, and causes of death with mortality rate, was tested by Chi-square test [9].

## Results and Discussion

The overall mortality rate was 10.93%, which varied from 1.10% (2006-07) to 25.56% (2004-05) (Table-1). The lower mortality rate in the particular years could be due to small number of kids born in that particular year, more kid born in favorable kidding season and better managemental conditions in comparison with the years having higher mortality. Different seasons of the year had a profound and significant effect (p<0.01, χ^2^=55.62) on mortality. Season wise mortality within the year indicated that the highest mortality was during rainy season (5.94%) followed by winter (3.24%) and summer (1.75%) season. Such fluctuation in survivability might be attributed to variation in climatic conditions and heavy incidences of some specific disease(s) in a particular season. Further in the rainy season mortality got aggravated due to high humidity, and high occurrence of coccidiosis. The overall mortality rate in our study was lower than reported [10] in cross-bred dairy goats (16.55%) at institutional farm. Awemu *et al*. [11] also reported a high rate of kids’ mortality in the wet season. However, Hailu *et al*. [12] reported lower survival rate for kids born in the dry season than those born in the wet season.

**Table-1:** Year and season-wise mortality rate in Rohilkhandi local goat.

Year	Summer (%)*	Rainy (%)*	Winter (%)*	Total (%)
2000-01	4.20 (5)	7.56 (9)	7.56 (9)	19.33 (23)
2001-02	3.95 (3)	9.21 (7)	9.21 (7)	22.37 (17)
2002-03	1.64 (2)	0.82 (1)	7.38 (9)	9.84 (12)
2003-04	2.33 (3)	17.83 (23)	4.65 (6)	24.81 (32)
2004-05	4.44 (4)	17.78 (16)	3.33 (3)	25.56 (23)
2005-06	0.90 (1)	12.61 (14)	0.00 (0)	13.51 (15)
2006-07	0.55 (1)	0.00 (0)	0.55 (1)	1.10 (2)
2007-08	1.49 (3)	7.96 (16)	1.00 (2)	10.45 (21)
2008-09	0.45 (1)	4.48 (10)	5.83 (13)	10.76 (24)
2009-10	2.13 (5)	6.81 (16)	2.13 (5)	11.06 (26)
2010-11	1.13 (4)	3.11 (11)	1.41 (5)	5.65 (20)
2011-12	1.84 (7)	2.36 (9)	3.15 (12)	7.35 (28)
Overall	1.75 (39)	5.94 (132)	3.24 (72)	10.93 (243)

Values in parenthesis indicate number of animals died during each season in each year.

* Significant (p≤0.01) (χ^2^=55.626)

The results also indicated that there is significant (p<0.05, χ^2^=16.083) association between age and season. The examination of age and season-wise mortality (Table-2) revealed that the mortality within 3 months of age is more in the 1^st^ month of age (2.34%) compared to next 2 months (1.35%). Thereafter, the mortality at early grower stage (4-6 months) was higher (2.52%) compared to late (7-12 months) grower stage (1.30%). Overall mortality after the age of 12 months (including very old and senile animals) was 3.42%. Season wise mortality within age group revealed that mortality in rainy was highest than other seasons in all age groups. In kids mortality during rainy season was highest in 0-1 month age group (1.26%) followed by 2-3 months age (0.81%). In growers, mortality was higher in 4-6 months (1.71%) age group compared to 7-12 months (0.63%) age group. Singh *et al*. [13] also reported highest pre-weaning mortality (69.03% of total kids’ mortality) occurred within 15 days of birth followed by 16-30 days. Similar results were also reported by Sabapara and Deshpande [14], Barbind and Dandewar [15], Singh *et al*. [16], and Ramachandran *et al*. [10] in different breeds.

**Table-2:** Age and season-wise mortality rate in Rohilkhandi local goat.

Age (month)	Summer (%)*	Rainy (%)*	Winter (%)*	Total (%)
0-1	0.31 (7)	1.26 (28)	0.76 (17)	2.34 (52)
2-3	0.09 (2)	0.81 (18)	0.45 (10)	1.35 (30)
4-6	0.13 (3)	1.71 (38)	0.67 (15)	2.52 (56)
7-12	0.31 (7)	0.63 (14)	0.36 (8)	1.30 (29)
>12	0.90 (20)	1.53 (34)	0.99 (22)	3.42 (76)
Overall	1.75 (39)	5.94 (132)	3.24 (72)	10.93 (243)

Values in parenthesis indicate number of animals died during each season in each age group.

* Significant (p≤0.05) (χ^2^=16.083)

The analysis of sex and season wise mortality it was found that the mortality rate in female (7.47%) was higher than the male (3.46%) animals (Table-3). The low mortality in male could be due to less availability at the farm at any given time as they were disposed from the farm after weaning. The mortality in both male and female were highest in rainy season (2.07% and 3.87%, respectively) followed by winter (0.99% and 2.25%, respectively) and summer (0.40% and 1.53%, respectively) season. Several reports support our finding that mortality in female is higher than male [17,18]. Mortality in males was reported higher in some findings [13,15]. No effect of gender on the mortality was observed. However, there are also few reports which suggest no effect of gender on mortality [19,20].

**Table-3:** Sex and season-wise mortality rate in Rohilkhand local goat.

Sex of animal	Summer (%)	Rainy (%)	Winter (%)	Total (%)
Male	0.40 (9)	2.07 (46)	0.99 (22)	3.46 (77)
Female	1.35 (30)	3.87 (86)	2.25 (50)	7.47 (166)
Overall	1.75 (39)	5.94 (132)	3.24 (72)	10.93 (243)

Values in parenthesis indicate number of animals died of particular sex during each season

The disease which singly contributed major share to overall mortality included digestive diseases (3.51%) followed by respiratory diseases (1.89%) and parasitic diseases (1.48%) (Table-4). Cardiovascular and musculoskeletal disorders contributed 0.63% and 0.18%, respectively to overall mortality. Mixed disorders (including any combination of either digestive, respiratory, parasite or cardiovascular diseases) contributed to 0.90% of mortality, and the remaining 2.34% of mortality was due to miscellaneous disorders in Rohilkhand local goats. Ramachandran *et al*. [10] also found that digestive disorders are primary cause of mortality followed by respiratory disorders. However, Bobde and Barbind [21] observed that mortality due to respiratory disease was significantly higher followed by digestive disease. Further, Bobde and Barbind [21] reported that enteritis in more in rainy season in Beetal × Osmanabadi kids.

**Table-4:** Causes and season wise mortality rate in Rohilkhand local goat.

Causes of death	Summer (%)	Rainy (%)	Winter (%)	Total (%)
Digestive disorder	0.58 (13)	1.93 (43)	0.99 (22)	3.51 (78)
Respiratory disorder	0.22 (5)	0.90 (20)	0.76 (17)	1.89 (42)
Cardiovascular disorder	0.18 (4)	0.27 (6)	0.18 (4)	0.63 (14)
Parasitic disorder	0.18 (4)	1.03 (23)	0.27 (6)	1.48 (33)
Musculo skeletal disorder	0.04 (1)	0.04 (1)	0.09 (2)	0.18 (4)
Mixed disorders	0.09 (2)	0.58 (13)	0.22 (5)	0.90 (20)
Miscellaneous disorder	0.45 (10)	1.17 (26)	0.72 (16)	2.34 (52)
Overall	1.75 (39)	5.94 (132)	3.24 (72)	10.93 (243)

Values in parenthesis indicate number of animals died due to each cause during each season

## Conclusion

This study confirms that overall mortality rate averaged 10.93% (ranged between 1.10% and 25.56%) over 12 years under semi-intensive farm condition. It was generally higher in rainy season followed by winter and summer. Season-wise mortality followed similar trend in all age groups. Mortality was higher in kids particularly under 1 month of age than in adults. The disease which singly contributed major share to overall mortality included digestive diseases followed by respiratory diseases and parasitic diseases.

## Authors’ Contributions

This study is part of Ph.D. special problem of the first author DU. MS and BHMP acted as advisory committee member and contributed immensely right from the start to end of the experiment. SS contributed in statistical analysis part. GKG contributed valuable suggestions during the experiment. All authors read and approved the final manuscript.
